# Protoconch enlargement in Western Atlantic turritelline gastropod species following the closure of the Central American Seaway

**DOI:** 10.1002/ece3.5120

**Published:** 2019-04-16

**Authors:** Stephanie Sang, Dana Suzanne Friend, Warren Douglas Allmon, Brendan Matthew Anderson

**Affiliations:** ^1^ Department of Earth and Atmospheric Sciences Snee Hall, Cornell University Ithaca New York; ^2^ Paleontological Research Institution Ithaca New York; ^3^ Present address: Department of Organismal Biology and Anatomy University of Chicago Chicago Illinois; ^4^ Present address: Department of Geology and Geography West Virginia University Morgantown West Virginia

**Keywords:** extinction selectivity, larval mode, macroevolution, Miocene, molecular phylogeny, Pliocene, *Turritella*

## Abstract

The closure of the late Neogene interoceanic seaways between the Western Atlantic (WA) and Tropical Eastern Pacific (TEP)—commonly referred to as the Central American Seaway—significantly decreased nutrient supply in the WA compared to the TEP. In marine invertebrates, an increase in parental investment is expected to be selectively favored in nutrient‐poor marine environments as prolonged feeding in the plankton becomes less reliable. Here, we examine turritelline gastropods, which were abundant and diverse across this region during the Neogene and serve as important paleoenvironmental proxies, and test whether species exhibit decreased planktotrophy in the WA postclosure as compared to preclosure fossils and extant TEP species. We also test for differences in degree of planktotrophy in extant sister species pairs. Degree of planktotrophy was inferred by measuring the size of protoconchs, the species' larval shell that represents egg size. Protoconch size was compared between extant postclosure WA and TEP species and preclosure fossil species. To compare extant sister species, we reconstructed the phylogeny of available WA and TEP species using one nuclear (H3) and three mitochondrial markers (12S, 16S, and COI). Compared to the preclosure fossils, protoconch size increased in WA species but remained the same in the TEP species. In the two extant sister species pairs recovered in the phylogenetic analysis, the WA species are inferred to be nonplanktotrophic while the TEP species are planktotrophic. This suggests that decreased nutrient availability and primary productivity in the WA may have driven this change in developmental mode, and was the primary selective force resulting in postclosure turritelline extinctions.

## 1 INTRODUCTION

The closure of the interoceanic seaways between the Tropical Eastern Pacific (TEP) and the Western Atlantic (WA)—commonly referred to as the Central American Seaway (O'Dea et al., 2018,2016)—in the late Neogene resulted in significant changes to the abiotic and biotic oceanographic conditions in the WA. Interoceanic connections for shallow‐water organisms, such as turritelline gastropods, persisted throughout the early stages of closure (Allmon, 2001; Beu, 2001; Coppard & Lessios, 2017; Hendy, 2013; Jackson & O'Dea, 2013; Lessios, 2008; Marko & Moran, 2002,2009; O'Dea et al., 2016), with communication of TEP upwelling waters to the WA through the middle Miocene (Anderson, Hendy, Johnson, & Allmon, 2017), shallowing around 8 Ma, but returning to deeper water communication by 6 Ma (Collins, 1996; Coates, Aubry, Berggren, Collins, & Kunk, 2003; Coates, Collins, Aubry, & Berggren, 2004; Leigh, O'Dea, & Vermeij, 2014; O'Dea et al., 2016). Final closure occurred between 3.5 and 2.7 Ma (Coppard & Lessios, 2017; Cronin & Dowsett, 1996; Jackson & O'Dea, 2013; Leigh et al., 2014; Molnar, 2008; O'Dea et al., 2018,2016).

As the interoceanic seaways closed, the WA experienced substantially reduced productivity (Allmon, 2001; Collins, 1996; O'Dea & Collins, 2013; Todd et al., 2002), due to some combination of reduced communication of Pacific upwelling waters (Anderson et al., 2017; Leigh et al., 2014; O'Dea et al., 2007), changes in circulation which may have reduced local upwelling (Allmon, 2001; Allmon, Emslie, Jones, & Morgan, 1996; Allmon, Rosenberg, Portell, & Schindler, 1996; Hays, Pisias, & Roelofs, 1989; Jackson & Budd, 1996; Jackson & O'Dea, 2013; Leigh et al., 2014; Lessios, 2008; Maier‐Reimer, Mikolajewicz, & Crowley, 1990; O'Dea et al., 2016; Todd et al., 2002), and possible decreased riverine nutrient input ~ 2.5 Ma (Aguilera et al., 2013; Pérez‐Consuegra et al., 2018). In response to decreased productivity, the biological community in the WA changed concurrently with this environmental change through shifts in the composition of benthic communities and life histories of benthic species (Jackson & Johnson, 2000; O'Dea et al., 2007; Todd et al., 2002), demonstrating the dramatic change in nutrient regime (Allmon, 1992,2001; Jackson & Johnson, 2000; Leigh et al., 2014; O'Dea et al., 2007,2016; Smith & Jackson, 2009; Todd et al., 2002). These ecological shifts were later (~1–2 Myr) accompanied by pulses of extinction, possibly due to declining population sizes (O'Dea et al., 2007; O'Dea et al., 2016).

In marine gastropods, larval mode is a life‐history trait that is predicted to have changed in response to declining WA nutrient productivity. Larval mode can generally be divided into two types based on whether larvae feed in the plankton: planktotrophic (feeding) or nonplanktotrophic (nonfeeding) (Jablonski & Lutz, 1980,1983; Thorson, 1950). These reproductive strategies result from trade‐offs between larval mortality and parental investment. Predation (Hickman, 2001) and starvation result in high larval mortality rates (as high as 99%) (Mileikovsky, 1971; Thorson, 1950). Larger, yolk‐rich eggs will reduce larval mortality, but higher parental investment results in fewer eggs being produced (Crisp & Spencer Davies, 1976; Jablonski & Lutz, 1980; Marshall, McAlister, & Retizel, 2018; Scheltema, 1971; Strathmann, 1978; Vance, 1973).

The closure of the Central American interoceanic seaways and associated changes in nutrient conditions in the WA allows us the opportunity to directly test the relationship between decreased ambient nutrient supply and nutrient apportionment in gastropod eggs. A decrease in marine nutrient supply is expected to result in decreased planktotrophy success and therefore favor increased parental investment (Fortunato, 2004; Jablonski & Lutz, 1980; Lessios, 1990,2008; Marshall et al., 2018; Miura, Frankel, & Torchin, 2011; Vance, 1973). Even if larvae still spend some time feeding in the plankton, larger offspring are better buffered against starvation and may need to spend less time in the plankton before settlement (Marshall & Keough, 2007; Marshall et al., 2018).

**Figure 1:**
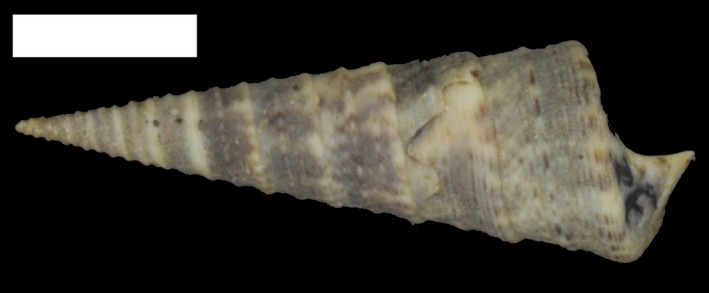
*Turritella banksii* (PRI 68087), a postclosure turritelline from the Tropical Eastern Pacific. Scale bar = 1 cm

We chose turritelline gastropods (Figure 1) to test our hypothesis that decreased nutrient availability selects for larger eggs. Turritelline gastropods are a highly diverse clade with as many as 150 valid Recent and ca. 800 valid fossil species and are often among the most abundant gastropods where they occur (Allmon, 1988,2011). Their typical affinity for fully marine environments coupled with their low trophic level has also led them to be important paleoclimate and environmental proxies (Allmon, 2011; Anderson et al., 2017; Jones & Allmon, 1995). Prior to the closure of the tropical American interoceanic seaways, turritellines were common and diverse in the WA (Allmon, 1992,2001; Anderson et al., 2017; Todd et al., 2002). Today, turritellines are rare in the modern WA and are represented by only three species: *T. exoleta* (Linnaeus, 1758), *T. variegata* (Linnaeus, 1758), and *T. acropora* (Dall, 1889). In contrast, the Late Miocene and Pliocene WA each contained over 25 species (Allmon, 1992; Allmon, Rosenberg, et al., 1996) and the Recent TEP is home to at least eight species: *T. anactor* (Berry, 1957), *T. banksii* (Reeve, 1849), *T. clarionensis*(Hertlein & Strong, 2011), *T. gonostoma*(Valenciennes, 1832), *T. leucostoma*(Valenciennes, 1832), *T. nodulosa*(King & Broderip, 1832), *T. radula*(Kiener, 1838), and *T. rubescens*(Reeve, 1849). These surviving WA lineages represent potential sister lineages (sometimes referred to as “geminate species”; Jordan, 1908; Marko & Moran, 2009; Miura et al., 2011) evolving separately for 3–5 Ma and under strikingly different nutrient regimes for at least 2 Ma (Todd et al., 2002; Todd & Johnson, 2013). The extinction of numerous WA turritelline species postclosure implies strong selective pressures on the WA species (Allmon, 1992).

Inferences can be made about the larval mode of fossil and extant gastropods based on observations of the protoconch (larval shell), which is sometimes retained at the apex of the adult shell (Fortunato, 2002,2004; Jablonski & Lutz, 1983; Jackson & Fortunato, 1996; Lima & Lutz, 1990; Shuto, 1974; Thorson, 1950; Vendetti, 2007). Large, paucispiral protoconchs are presumed to be formed by larval gastropods that have spent little or no time in the plankton, and narrow, multispiral protoconchs are thought to indicate prolonged planktonic phases. Shuto (1974) used living taxa of known larval mode to describe what quantitative values in protoconch maximum diameter and number of volutions are associated with each mode. Although egg size, as inferred from protoconch size, is not the sole form of increased parental investment (e.g., it does not capture different nutritional content in an egg), it is a useful approximation (Marshall et al., 2018; Moran & McAlister, 2009).

It is expected that decreased nutrient availability in the plankton would result in selection for greater nutrient apportionment (larger eggs) which should be reflected in increased sizes for protoconchs in postclosure WA species. We test these hypotheses by comparing (a) turritelline protoconch size in the postclosure WA with both modern TEP protoconch sizes and preclosure fossil protoconch sizes, and (b) comparing protoconch size changes in extant sister species pairs by generating a robust molecular phylogeny of TEP and WA turritellines based on H3, COI, 16S, and 12S sequence data.

## 2 MATERIALS AND METHODS

### 2.1 Taxon sampling

We sampled seven of the eight species of *Turritella* in the TEP and two of the four species in the WA (Table 1). Protoconch preservation can be rare, even in live‐collected individuals from modern species. Preclosure WA fossil species examined are those described from the late Miocene of Panama and the late Oligocene of Venezuela. Although the only protoconch sampled for the extant species *Vermicularia knorrii* (Deshayes, 1843) was from a Pleistocene specimen, the specimen is considered part of the postclosure WA fauna. *Batillaria zonalis* (Bruguière, 1792) and *Lampania cumingi* (Gray, 1847) (Batillariidae) were selected as out‐groups due to the consistent placement of Batillaridae as sister to Turritellidae (Strong et al. 2011).

**Table 1:** Taxa examined in this study

Species	Distribution	16S	COI	12S	H3
*T. acropora*	Cuba, Mexico (Dall, 1889)	MK368669 MK368670 M94001.1	MK368688 MK368689	MK527210 MK527211	MK513802 MK513803
*T. altilira* a	Panama, Colombia, Venezuela. Miocene. (Woodring, 1957)	n/a	n/a	n/a	n/a
*T. anactor*	Gulf of CA to Puerto Penasco, Sonora, Mexico (Keen, 1971)	M94002.1	–	–	–
*T. bacillum*	South‐east Asia (Kiener, 1838)	MK368671 MK368672	–	MK527212 MK527213	MK513804 MK513805
*T. banksii*	Guaymas, Mexico, to Ecuador (Keen, 1971)	MK368673 MK368674	MK386466 MK368699	MK527214 MK527215	MK513816
*T. bifastigata* a		n/a	n/a	n/a	n/a
*T. broderipiana*	Peru (d'Orbigny, 1835)	–	–	–	–
*T. clarionensis*	Gulf of CA to Panama (Keen, 1971)	–	–	–	–
*T. exoleta*	Gulf of Mexico (Linneaus, 1758)	MK368679 MK368680 MK368681 M94004.1	MK368693 MK368694	MK527217 MK527218	MK513808 MK513809 MK513810
*T. gatunensis* a	Panama, Colombia, Venezuela (Conrad, 1857)	n/a	n/a	n/a	n/a
*T. gonostoma*	Gulf of CA to Ecuador (Keen, 1971)	MK368682 M94005.1	–	MK527219	–
*T. leucostoma*	Gulf of CA to Panama (Keen, 1971)	M94006.1	MK368695	MK527220	MK513811
*T. mariana = T. radula*	Gulf of CA to southern Colombia (Keen, 1971)	n/a	n/a	n/a	n/a
*T. matarucana* a	Panama, Colombia, Venezuela (Hodson, 1926)	n/a	n/a	n/a	n/a
*T. nodulosa*	Baja, CA, to Southern Gulf of CA, to south of Ecuador (Keen, 1971)	MK368684 MK368685 M94007.1	MK368696 MK368700	MK527221 MK527222	MK513812 MK513813
*T. radula*	Pacific, Gulf of CA to Colombia (Keen, 1971)	MK368677 MK368678	MK368690 MK368691	–	MK513806 MK513807
*T. rubescens*	San Francisco Island, Gulf of CA, to Gorgos Island, Colombia (Keen, 1971)	MK368686	MK368697	MK527223	MK513814
*T. terebra*	Taiwan, China (Linneaus, 1758)	M94008.1 MK368687	MK368698	MK527224	–
*T. variegata*	Cuba, Puerto Rico, Jamaica, Colombia, Venezuela (Mioslavich et al. 2010)	–	–	–	–
*T. venezuelana* a	Venezuela (Hodson, 1926)	n/a	n/a	n/a	n/a
*T. willetti*	Pacific (McLean, 1970)	–	–	–	–
*Vermicularia knorrii*	Gulf Coast, Antigua, Barbuda, Cuba, Jamaica, Florida to North Carolina, Bermuda (Deshayes, & Milne‐Edwards, 1843)	n/a	n/a	n/a	n/a
*Vermicularia woodringi* a	Florida, North Carolina, South Carolina, c.f. Jamaica (Olsson and Harbison, 1953)	n/a	n/a	n/a	n/a
*Batillaria zonalis*	Japan, Korea, China (Bruguière, 1792; Miura et al. 2005)	HQ833976.1	AB211356.1	HQ833856.1	HQ834127.1
*Lampania cumingi*	Japan, Korea, China, invasive to Pacific Northwest of the US (Miura et al. 2005)	HQ833975.1	HQ709375.1	HQ833855.1	HQ834126.1

Note

GenBank accession numbers for sequence data listed for each marker examined.

a Denotes extinct taxa.

Wet specimens were obtained from the collections of the Florida Museum of Natural History (FLMNH) and Paleontological Research Institution (PRI). Specimens of *T. banksii* were collected at Bique, Panama. Protoconch data were obtained from specimens in the collections of the FLMNH, PRI, and Academy of Natural Sciences (ANSP), as well as from material collected in April 2014 at various localities in Colón, Panama. Specimens newly collected for this analysis are stored at the PRI, and DNA elutions are archived at the Cornell Lab of Ornithology. In molecular analyses, data sources are identified as UF = University of Florida, FLMNH collection, S = this study collected, and L = Lieberman, Allmon, and Eldredge (1993) from GenBank data.

### 2.2 DNA extraction, sequencing, and alignment

Genomic DNA was extracted using the Qiagen DNeasy Kit from about 100 mg of tissue, following the manufacturer's protocol. We chose the mitochondrial 16S, 12S, cytochrome c oxidase subunit I (COI), and nuclear histone H3 regions for sequencing because 16S fragments are available from a subset of our species (Lieberman et al., 1993), and because there exist gastropod‐specific primers for these genes (Miura, Torchin, Kuris, Hechinger, & Chiba, 2006; Simon, Franke, & Martin, 1991; Zou, Li, & Kong, 2011) (Table 2). The PCR mixture included 0.002 μg/μl bovine serum albumen to improve PCR yields (Farell & Alexandre, 2012; Woide, Zink, & Thalhammer, 2010). Each reaction ran for 35 cycles of 95°C for 4.5 min, 95°C for 1 min, between 54 and 64°C for 1 min (the optimal annealing temperature varied), 72°C for 1:20 min, and 72°C for 4.5 min. In preparation for sequencing, all PCR products were treated with exonuclease (10 U/μl) and shrimp alkaline phosphatase (1 U/μl) at 37°C for 30 min and then at 90°C for 10 min. Sanger sequencing took place at the Cornell Biotechnology Resource Center. Newly sequenced molecular data were then aligned with previously published GenBank data (Table 1). Sequences were aligned using MAFFT‐L‐INS‐i v. 7 (Katoh & Standley, 2013) and checked with Mesquite v. 3.0.3 (Maddison & Maddison, 2015) by eye. Genes were concatenated with SequenceMatrix v. 1.7.8 (Vaidya, Lohman, & Meier, 2011). Mesquite was then used to annotate codon positions.

**Table 2:** Primer pairs for each gene region

Gene region	Forward primer	Reverse primer	Length
16S	16Sar: 5' CGC CTG TTT ATC AAA AAC AT 3' (Simon et al. 1991)	16Sbr: 5' CCG GTC TGA ACT CAG ATC ACG T 3' (Simon et al. 1991)	527 bp
COI (1st half)	COIbf: 5' GGG GCT CCT GAT ATA GCT TTT CC 3' (Miura et al. 2006)	COIbrINT: 5' GCA TAA ATT ATC CCT AAA GTC CC 3' (this study)	969 bp
COI (2nd half)	COIbfINT: 5' TTC TTC CTG GGT TTG GGA TAA TCT C 3'(this study)	COIbr: 5' TAA TAT AGA AGT GTG CTT TAG T 3' (Miura et al. 2006)	
12S	12SF: 5' AAA GCT TCA AAC TGG GAT TAG ATA CCC CAC TAT 3' (Zou et al. 2011)	12SR: 5' TGA CTG CAG AGG GTG ACG GGC GGT GTG T 3' (Zou et al. 2011)	456 bp
H3	H3NF: 5' ATG GCT CGT ACC AAG CAG AC 3' (Colgan et al. 1998)	H3NR: 5' ATR TCC TTG GGC ATG ATT GTT AC 3' (Colgan et al. 1998)	376 bp

### 2.3 Phylogenetic analysis

Phylogenetic analysis of molecular characters was performed with parsimony, maximum‐likelihood, and Bayesian methods. Parsimony analysis was run using PAUP* v. 4.0a141 (Swofford, 2002). Out of 2,328 total characters, 558 were parsimony‐informative. Overall base pair frequencies were calculated as A = 0.28, T = 0.30, C = 0.21, G = 0.21. A heuristic search was set with random stepwise addition (10,000 repetitions) and a TBR swapping algorithm. All other settings were left as default.

For maximum‐likelihood analysis, sequences were partitioned by codon position in each gene and run under a GTRCAT (default setting) model with joint branch length optimization using RAxML 8.0.9 on the Cyberinfrastructure for Phylogenetic Research (CIPRES) platform (Miller, Pfeiffer, & Schwartz, 2010) to calculate the ML tree and nonparametric bootstrap node support. The resulting tree was visualized on FigTree v. 1.4.2 (Rambaut, 2016).

For Bayesian analysis, sequences were entered into MrBayes v. 3.4.2 (Ronquist & Huelsenbeck, 2003) on the CIPRES system. Each gene was partitioned by codon position. The partitions were assigned a model of best‐fit in PartitionFinder v. 1.1.1 (Lanfear, Calcott, Ho, & Guindon, 2012) according to the Akaike information criterion (Table S1). In MrBayes, two runs were conducted with four chains each for 10 million generations. The first 25% of results were discarded as burn‐in. All other settings were left as default. Log files were combined and checked with Tracer v. 1.6 (Rambaut & Suchard, 2014). A statistical summary of the ML and Bayesian analyses is presented in Table S2.

### 2.4 Protoconch measurements

Specimens with intact protoconchs were almost entirely found on juveniles less than one centimeter in length. Protoconchs are often abraded away in turritellines, even during the life of the organism (Johnson, Anderson, & Allmon, 2017). The protoconch is composed of two parts: protoconch I, which is the embryonic shell, formed prior to hatching and is unornamented, and protoconch II which is produced prior to metamorphosis, and which may be smooth or ornamented (Jablonski & Lutz, 1983; Robertson, 1971). Whole shells were sputter‐coated with a thin layer of gold then imaged on a scanning electron microscope (JCM‐6000 NeoScope Benchtop SEM) at the PRI. Venezuelan specimens from the type and figured collection of the PRI were imaged without sputter‐coating. Side and top view images were taken to identify the protoconch I–protoconch II boundary, which was then marked on the top view image. We used this boundary to find the total number of volutions (full 360‐degree spirals) in protoconch I. The diameter of protoconch I was measured at its widest using ImageJ v.1.45s (Schneider, Rasband, & Eliceiri, 2012).

### 2.5 Analysis of protoconch character divergence

Statistical comparisons were made among protoconch data obtained from preclosure fossil, postclosure Atlantic, and postclosure Pacific specimens in Past3 (Hammer, Harper, & Ryan, 2001). Both protoconch maximum diameter and diameter/volutions ratio were compared. Tukey's *Q* was calculated to make comparisons among means for all three data sets simultaneously, with significance estimated according to the method of Copenhaver and Holland (1988). The Mann–Whitney *U* test was applied to determine whether the samples were likely to be drawn from the same distributions.

Continuous character mapping of protoconch diameters on the molecular phylogeny was achieved using the “contMap” function in the “phytools” (Revell, 2012) package for R. The “contMap” function estimates character states at internal nodes using ML methods (function “anc.ML”). From the Bayesian tree, multiple individuals for each species were collapsed into one tip using the “delete subelements” function in TreeGraph2 (v. 2.14.0‐771) (Stöver & Müller, 2010) to create a consolidated backbone. The average protoconch diameter for each species was then mapped onto each tip.

## 3 RESULTS

### 3.1 Molecular phylogeny

**Figure 2:**
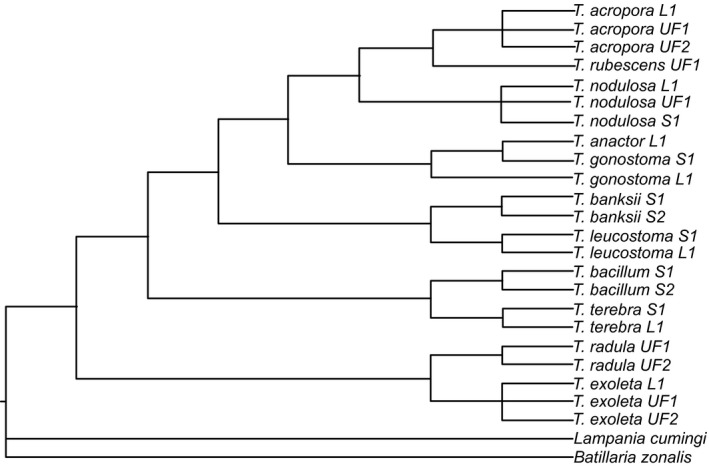
Majority rule parsimony tree (consensus of 81 trees) generated from mitochondrial and nuclear sequences. All species are from genus *Turritella,* except for out‐groups *Batillaria zonalis* and *Lampania cumingi*. L1 = sequence from Lieberman et al. (1993); S1 or S2 = specimen collected for this study; UF1 or UF2 = specimen from FLMNH

**Figure 3:**
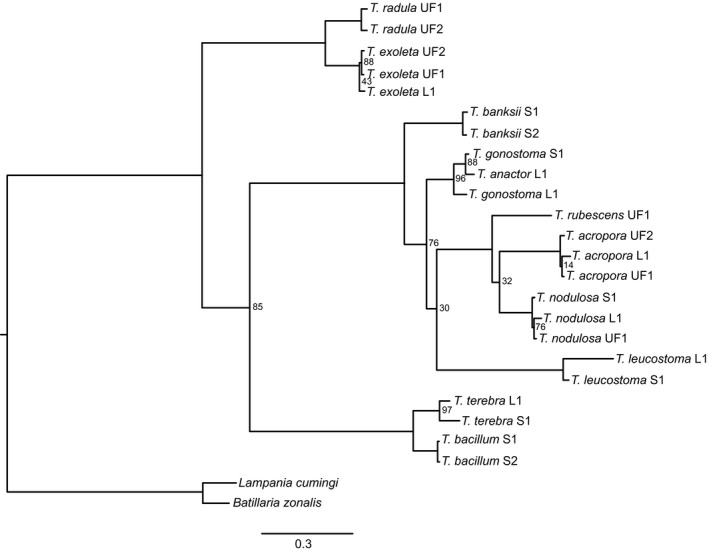
Maximum‐likelihood tree generated from mitochondrial and nuclear sequences. Unless noted, bootstrap values at each node are 100. All species are from genus *Turritella*, except for out‐groups *Batillaria zonalis* and *Lampania cumingi*. L1 = sequence from Lieberman et al. (1993); S1 or S2 = specimen collected for this study; UF1 or UF2 = specimen from FLMNH. Scale bar represented mean number of nucleotide substitutions per site

**Figure 4:**
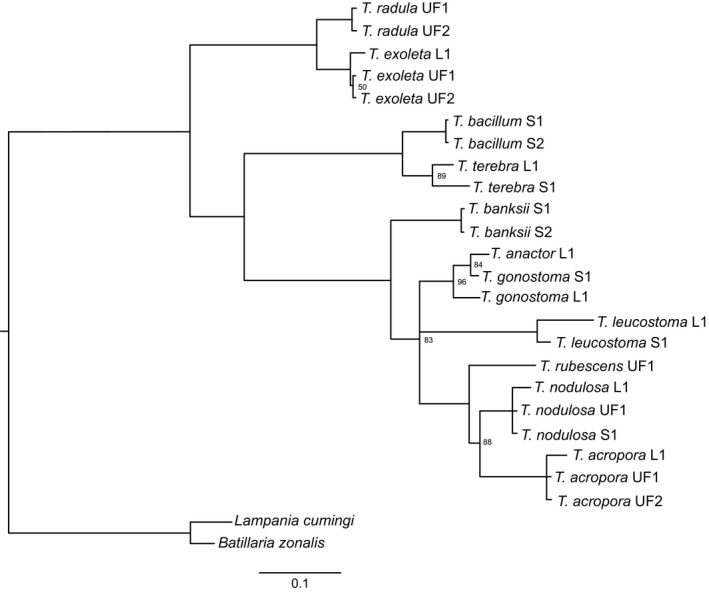
Bayesian tree generated from nuclear and mitochondrial sequence data. Posterior probabilities at nodes are 100 unless noted. All species are from genus *Turritella,* except for out‐groups *Batillaria zonalis* and *Lampania cumingi*. L1 = sequence from Lieberman et al. (1993); S1 or S2 = specimen collected for this study; UF1 or UF2 = specimen from FLMNH

Two extant sister species pairs are consistently identified in the molecular trees (Figures 2, 3, 4). The first pair is *T. exoleta* (WA) and *T. radula* (TEP), which was discovered in all three methods (parsimony, maximum‐likelihood, and Bayesian). The second pair, *T. acropora* (WA) and *T. nodulosa*(TEP), is identified in both the maximum‐likelihood and Bayesian results (Figures 3 and 4), but exists as a “sister species cluster” with *T. rubescens* under the parsimony method (Figure 2).

All methods find three major clades within these turritellines: (a) *T. exoleta* and *T. radula* sister to all other taxa, (b) *T. bacillum* and *T. terebra* sister to the remaining taxa, and (c) all other species. Most of the incongruence is located within this last clade due to unstable placement of *T. banksii, T. leucostoma*, and *T. rubescens* among methodologies.

### 3.2 Protoconch size changes after closure of the Central American Seaway

**Figure 5:**
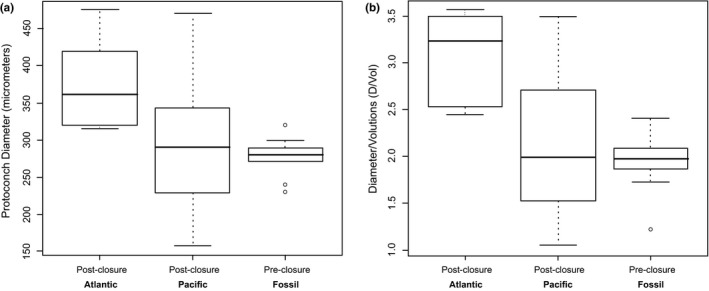
Postclosure Atlantic species are nonplanktotrophic compared to planktotrophic postclosure Pacific and preclosure fossil species. Quartile plots of (a) protoconch diameter and (b) diameter/volutions (D/Vol) ratio for postclosure Atlantic, postclosure Pacific, and preclosure fossil species

Protoconch size data were obtained for the species identified in Table 3. We found that postclosure WA turritelline species as a whole experienced significant change in both protoconch diameter (Tables 4 and 5) and in diameter/volutions (D/Vol) compared with preclosure values (Figure 5; Tables 4 and 6). No significant change was found in TEP species relative to the preclosure fossil species in maximum diameter (Table 5) or diameter/volutions ratio (Table 6). Protoconch diameters (Tables 5) and D/Vol (Table 6) measurements differ significantly between Recent WA and Recent TEP species.

**Table 3:** Turritelline protoconch diameters and diameter/volutions ratios observed in this study

Species	Cohort	Age	Diameter (μm)	Diameter/volutions
*Turritella acropora*	Postclosure Atlantic	Recent	475	3.17
*T. acropora*	Postclosure Atlantic	Recent	420	2.44
*T. altilira*	Preclosure fossil	Miocene	276	2.40
*T. altilira*	Preclosure fossil	Miocene	272	2.18
*T. altilira*	Preclosure fossil	Miocene	300	1.23
*T. anactor*	Postclosure Pacific	Recent	316.11	2.70
*T. banksii*	Postclosure Pacific	Recent	285.57	2.72
*T. broderipiana*	Postclosure Pacific	Recent	426.5	3.23
*T. exoleta*	Postclosure Atlantic	Recent	350	3.50
*T. exoleta*	Postclosure Atlantic	Recent	373.49	3.29
*T. gatunensis*	Preclosure fossil	Miocene	282	1.97
*T. gatunensis*	Preclosure fossil	Miocene	290.354	1.98
*T. gonostoma*	Postclosure Pacific	Recent	407	2.04
*T. gonostoma*	Postclosure Pacific	Recent	470	2.35
*T. leucostoma*	Postclosure Pacific	Recent	274.19	2.49
*T. leucostoma*	Postclosure Pacific	Recent	269.66	1.80
*T. leucostoma*	Postclosure Pacific	Recent	230	1.50
*T.radula (“mariana”)*	Postclosure Pacific	Recent	203.53	1.36
*T. nodulosa*	Postclosure Pacific	Recent	228.15	1.95
*T. nodulosa*	Postclosure Pacific	Recent	299.662	1.65
*T. nodulosa*	Postclosure Pacific	Recent	294.197	1.56
*T. nodulosa*	Postclosure Pacific	Recent	300	1.50
*T. radula*	Postclosure Pacific	Recent	167.238	1.67
*T. radula*	Postclosure Pacific	Recent	161.274	1.38
*T. radula*	Postclosure Pacific	Recent	157.506	1.05
*T. rubescens*	Postclosure Pacific	Recent	256.643	2.02
*T. willetti*	Postclosure Pacific	Recent	348.211	3.48
*T. willetti*	Postclosure Pacific	Recent	341	3.04
*T. wiilletti*	Postclosure Pacific	Recent	346.6	2.84
*T. exoleta*	Postclosure Atlantic	Recent	320.62	3.56
*T. variegata*	Postclosure Atlantic	Recent	316.17	2.53
*T. venezuelana*	Preclosure fossil	Late Oligocene	230	1.73
*T. gilbertharrisi*	Preclosure fossil	Late Oligocene	240	1.92
*Vermicularia knorrii*	Postclosure Atlantic	Pleistocene	320	2.09
*V. woodringi*	Preclosure fossil	Miocene	280	1.87

**Table 4:** Comparison of turritelline protoconch diameter among preclosure (late Oligocene–middle Miocene) fossil, postclosure (Pleistocene–Recent) Atlantic, and postclosure Pacific species

	Preclosure fossil	Postclosure Atlantic	Postclosure Pacific
*N* (protoconchs)	8	7	20
*N* (species represented)	5	4	10
Min (μm)	230	316.2	157.5
Max (μm)	300	475	470
Mean (μm)	271.3	367.9	289.2
*SE*	8.5	22.8	19.2
Variance	583.6	3623.8	7404.5
*SD*	24	60.2	86.0
Median	278	350	289.9

**Table 5:** Statistical comparisons of protoconch diameter among preclosure fossil, postclosure Atlantic, and postclosure Pacific turritellines

Protoconch diameter	Tukey's *Q*	Mann–Whitney *U*
Preclosure fossil versus Postclosure Atlantic	4.114; *p* = **0.01758**	0; *p* = **0.004**
Preclosure fossil versus Recent Pacific	0.7605; *p* = 0.8534	69; *p* = 1
Pacific versus Postclosure Atlantic	3.354; *p* = 0.06041	26; *p* = **0.048**

Note

Statistically significant *p* values in bold.

**Table 6:** Statistical comparisons of protoconch diameter/volutions ratios among preclosure fossil, postclosure Atlantic, and postclosure Pacific turritellines

Protoconch D/Vol	Tukey's *Q*	Mann–Whitney *U*
Preclosure fossil versus Postclosure Atlantic	5.145; *p* = 0.002695	2; *p* = **0.008386**
Preclosure fossil versus Recent Pacific	1.429; *p* = 0.5756	50; *p* = 1
Recent Pacific versus Postclosure Atlantic	3.716; *p* = 0.03385	38; *p* = **0.0204**

Note

Statistically significantly values in bold.

**Figure 6:**
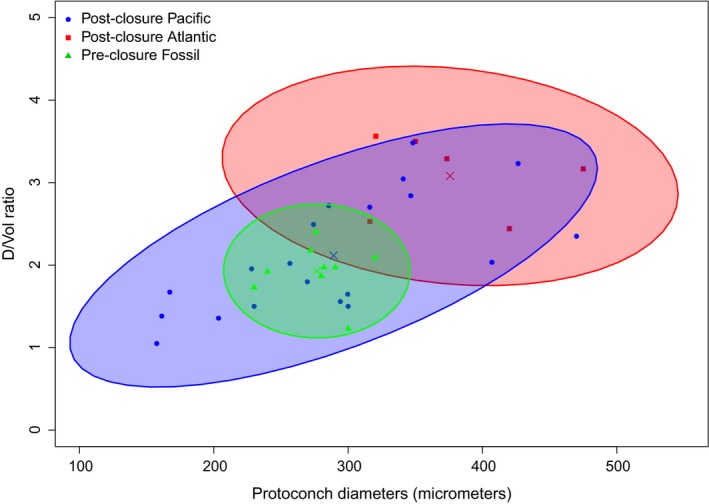
Changes in character space occupied by preclosure fossil, and postclosure Atlantic and postclosure Pacific turritelline protoconchs. 90% confidence ellipses are shown

The distribution of these measurements is shown in Figure 6, where all observed protoconch diameters are plotted against D/Vol. Distributions of fossil and WA protoconch characteristics occupy different areas in component space, whereas the distribution of TEP protoconch characteristics is an expansion of the fossil distribution.

## 4 DISCUSSION

### 4.1 Evolution of developmental mode in Central American Isthmus turritellines

We find evidence of increased nonplanktotrophy in WA species and conclude that this was likely a response to decreased nutrient availability in the WA after the closure of the interoceanic seaways. The similarity of preclosure protoconch diameters to postclosure TEP protoconch diameters affirms that the observed difference in the modern populations is not due to a decline in mean protoconch size in the Pacific. The minimum protoconch size observed (157.5 μm) was from a TEP species and is nearly half of the minimum observed size for WA species (316.2 μm). Maximum observed protoconch sizes were similar between modern WA (475 μm) and TEP (470 μm) species. This indicates that selection against small protoconch size was the likely driver of this change. Because we show that the WA species examined are sister to EP species in our molecular phylogeny, we regard the evolution of increased nonplanktotrophy as separate, independent occurrences within each lineage. The observation that these surviving lineages have independently increased protoconch sizes relative to preclosure along with the observation of similar changes in other taxa (Fortunato, 2004; Jackson, Jung, & Fortunato, 1996; Lessios, 1990; Miura et al., 2011; Moran, 2004; Wehrtmann & Albornoz, 2002) strongly supports the adaptive significance of these changes.

This phylogeny updates the only existing molecular phylogeny of turritellines that was based only on partial mitochondrial 16S sequences (Lieberman et al., 1993). We recover a different topology than presented in Lieberman et al. (1993) due to the additional genetic data; we double the read length for the 16S sequences and add in three other genes to our dataset. In that previous study, which included many of the same species, turritellines were used as a case study to investigate whether there were signals of species selection favoring increased diversification of nonplanktotrophic species. This hypothesis was motivated by the observation that nonplanktotrophic turritelline species outnumber planktotrophic species approximately 3:1 in the Neogene Gulf Coastal Plain, and worldwide today nonplanktotrophic species are twice as common as planktotrophic species (Allmon, 1992). Larval mode has been considered to be a particularly important factor for macroevolution and speciation rates in gastropods (Crampton, Cooper, Beu, Foote, & Marshall, 2010; Hansen, 1978; Jablonski, 1987; Jablonski & Valentine, 1990; Krug et al., 2015; Parsons, 1997; Scheltema, 1971,1978). Planktotrophic larvae tend to spend more time in the plankton and therefore generally have higher dispersal potential, wider geographic ranges, and lower rates of isolate formation and consequent speciation. Nonplanktotrophic species, which spend little or no time in the plankton, generally have decreased dispersal, narrower ranges, and consequently, higher theoretical potential for allopatric speciation and extinction (Bhaud, 1993; Jablonski & Lutz, 1980,1983; Jackson et al., 1996; Vermeij, 1982). While the Lieberman et al. (1993) topology is markedly different from the ones shown in this paper, both studies indicate that the ancestral turritelline condition was planktotrophy. This conclusion is further strengthened for the observed taxa by our finding that all preclosure forms in the Central American Isthmus region had protoconch sizes indicative of planktotrophy. Additionally, both this paper and Lieberman et al. (1993) find that nonplanktotrophy arose within single species instead of at the base of nonplanktotrophic clades, and so likely did not drive increased speciation in the sampled taxa.

**Figure 7:**
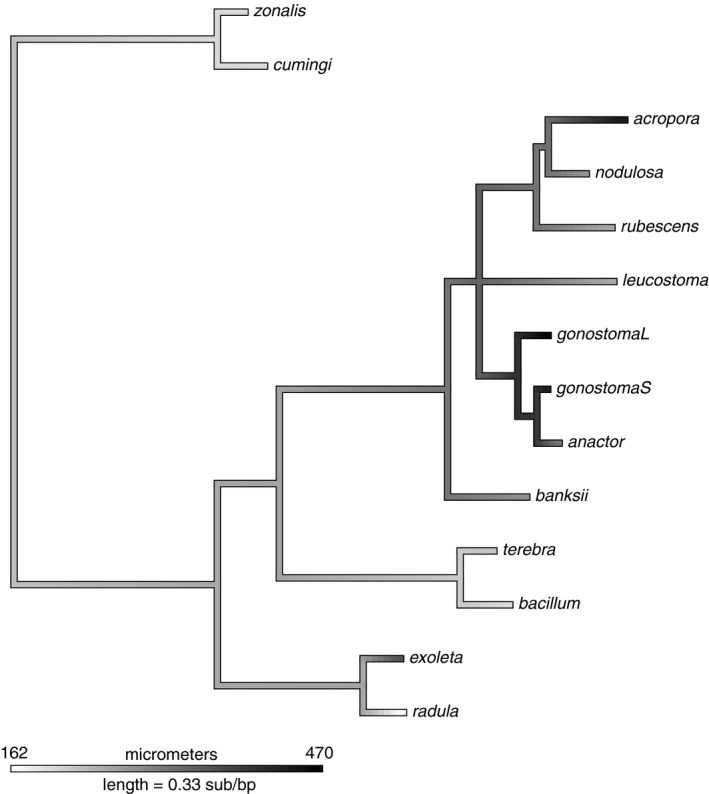
Ancestral state reconstruction with the trait value (grayscale) indicating protoconch diameter (micrometers), plotted onto a consolidated Bayesian phylogenetic tree. Our results indicate that the ancestral turritelline was likely planktotrophic. The “L” and “S” in *T. gonostoma* indicate whether the sequence was from Lieberman et al. (1993) or collected for this study, respectively. Length of the legend (=0.33) represents the length of the legend in units of branch length, which is expected mutations per site

The pattern of increased protoconch size in postclosure WA species is consistent with our hypothesis that decreased nutrient availability in the WA selected for nonplanktotrophy, and the phylogeny indicates that at least two of the three Recent WA species evolved larger protoconch sizes independently (Figure 7). Further research is, however, necessary to determine the underlying macroevolutionary mechanisms responsible for these changes. As modern TEP turritellines exhibit a great diversity of protoconch sizes, it is possible that the modern differences are the result of either selective extinction of lineages which have small protoconchs (inferred planktotrophs, following Shuto, 1974), or selection on each lineage for larger protoconchs through time in the WA, with possible unrelated extinctions. The presumed difficulty of re‐evolving planktotrophy also may bias the long‐term accumulation of nonplanktotrophy in a clade, if the transition to nonplanktotrophy has no consequences for speciation (Duda & Palumbi, 1999; Krug et al., 2015). There are two chief difficulties in assessing which of these macroevolutionary mechanisms was involved in the transition to larger protoconch sizes in WA turritellines. First, additional fossil protoconch data would need to be incorporated into a phylogenetic framework to distinguish between these evolutionary histories. Data from additional protoconchs, with both high‐resolution stratigraphic data and confident species assignments, are obviously vital to assess the possibility of anagenetic selection for protoconch size increase. Efforts should be made to document protoconch sizes in the literature where possible, even maximum diameters from fragmented protoconchs, and collecting efforts should take special care not to neglect small apical fragments which may be rapidly screened for protoconchs using light microscopy. Second, the present status of turritelline systematics presents a further difficulty. It has been the operational assumption of many studies that long‐distance dispersal events among turritellines are rare (e.g., Marwick, 1957). Both Lieberman et al. (1993) and the present study suggest that this assumption should be treated with some caution as there appear to be two clades in the neotropics, one of which is sister to a clade of species from South‐East Asia. A global molecular phylogeny of Recent turritellines is needed to assess the validity of this assumption in regard to fossil species from the tropical Americas, and to aid in determining what morphological characters may be informative in assigning species to these clades.

Regardless of the evolutionary mechanisms involved in achieving nonplanktotrophy, decreased planktotrophy and diversity of turritellines after the closure of tropical American interoceanic seaways will likely have long‐term consequences for the evolution of turritellines. Species‐poor clades are more likely to be subject to stochastic extinction, and low‐dispersal larvae may result in shifts in speciation rates, or, in punctuational systems, shifts in rates of morphospace exploration (Jablonski, 2017; Krug et al., 2015). Our phylogeny indicates that the two WA species examined are not closely related, and therefore, loss of either would substantially decrease the phylogenetic diversity (Faith, 1992) present in the region. The loss of planktotrophy has also been considered subject to Dollo's law, with limited opportunities for reversal due to the complex of characters necessary for larval feeding (Krug et al., 2015). This may not be the case as even direct‐developing gastropods may pass through a veliger stage within the egg, without loss of associated characters (e.g., larval velum; Collin, 2004; Collin, Chaparro, Winkler, & Veliz, 2007; Collin & Cipriani, 2003; Collin & Miglietta, 2008). If increased nonplanktotrophy decreases net diversification rates, selection toward higher parental investment in WA turritelline clades may have contributed to the overall decline in WA diversity as well (Krug et al., 2015). Investigating the evolution of WA and TEP turritelline protoconch size in a phylogenetic context may distinguish whether nonplanktotrophy has led to decreased net diversification rates in WA turritellines. If such a decrease is observed, then this shift in protoconch sizes may be evidence that WA turritellines represent two “dead clades walking” (Jablonski, 2002; Krug et al., 2015) following the Pliocene extinctions.

## CONFLICT OF INTEREST

None declared.

## AUTHOR CONTRIBUTION

Stephanie Sang drafted the initial manuscript, performed molecular analyses, collected specimens, and performed analysis of both modern protoconchs and fossil protoconchs from Panama. Brendan Anderson led manuscript writing, performed Venezuelan protoconch analysis, and performed statistical analyses. Dana Friend assisted with identification and measurement of specimens and discussion of gastropod protoconch literature. Warren Allmon conceived of and supervised the project and edited the manuscript.

## Data Availability

All molecular sequence data used in our analyses are available in GenBank, as outlined in Table 1.
