# Supplementary material for: Protoconch enlargement in Western Atlantic turritelline gastropod species following the closure of the Central American Seaway
Source: Ecol Evol. 2019 Apr 16;9(9):5309–23. doi: 10.1002/ece3.5120 (PMC6509377; doi:10.1002/ece3.5120)
Supplement: Supplementary file 1 [file ECE3-9-5309-s001.docx]

**Supplementary Information**

**Table S1:** Substitution models used in Bayesian analysis

| **Partition** | **Model** |
| --- | --- |
| **16S - Pos 1** | GTR+I+G |
| **16S - Pos 2** | GTR+I+G |
| **16S - Pos 3** | GTR+I+G |
| **COI - Pos 1** | GTR+G |
| **COI - Pos 2** | HKY |
| **COI - Pos 3** | HKY+G |
| **12S - Pos 1** | GTR+I+G |
| **12S - Pos 2** | GTR+I+G |
| **12S - Pos 3** | GTR+I+G |
| **H3 - Pos 1** | F81 |
| **H3 - Pos 2** | JC+I |
| **H3 - Pos 3** | GTR |

**Table S2:** Summary of Maximum Likelihood and Bayesian analysis

| **Method** | **No. Char** | **No. Tips** | **No. Gen.** | **Harmonic Mean** |
| --- | --- | --- | --- | --- |
| Bayesian | 2328 | 26 | 10M | -8913.90 |

| **Method** | **No. Char** | **No. Tips** | **No. Gen.** | **Final ML Likelihood** |
| --- | --- | --- | --- | --- |
| Maximum Likelihood | 2328 | 26 | 10M | -8870.55 |
